# Supplementary material for: Creatine kinase and neuromuscular fatigue responses following differing spells of simulated cricket fast bowling
Source: PLoS One. 2025 Jan 22;20(1):e0317692. doi: 10.1371/journal.pone.0317692 (PMC11753691; doi:10.1371/journal.pone.0317692)
Supplement: Attachment — Submitted filename: Response to Reviewers for CK and NMF fast bowling.docx [file pone.0317692.s003.docx]

12^th^ June 2024

PONE-D-24-16732; Creatine Kinase and Neuromuscular Fatigue Responses Following Differing Spells of Simulated Cricket Fast Bowling

Dear Reviewer(s),

Firstly, we would like to thank you very much for taking the time to review our original research article for consideration for publication within the ‘PLOS ONE’ journal.

Below we address each of the individual points raised:

**Overall:**

- Thank you very much for the kind words pertaining to this research area and our understanding of the literature.
- We acknowledge the length of this paper and have worked to ensure this is clear and concise and that it now reads as a journal article versus a thesis.

**Abstract:**

- P2L32: grammatical error revised
- PlayerLoad has been repositioned earlier

**Introduction:**

- Thank you again for the useful and constructive feedback. Substantial sections of the introduction have been edited to condense the text ensuring a concise yet comprehensive assessment of the current literature allows the reader to understand the requirement for the study and how and why the aim and hypothesis has been developed. The two specific line examples identified in the reviewer comments have not been referenced here as the changes have been incorporated into the larger changes made to the section.

**Method:**

- P7L159: What makes these bowlers highly skilled? – This statement has been removed.
- Subjects has been changed to participants throughout.
- I take it your data was normally distributed as you ran an ANOVA instead of a Kruskal Wallis test? The same as a t-test instead of a Wilcoxon signed-rank test? If this is the case then it needs to be stated in your data analysis section. This information has now been included in the method section of the paper
- P10L226-233: I feel this needs to come into the methods earlier on. I was quite confused as to what the CA-AIS was until I got to this paragraph. This has been amended in the general re-organisation of the methods section.

**Results:**

- All tables have been re-formatted to make them easier to read and ensure that spacing is evenly distributed.
- Additionally, a substantial portion of the text has now been removed directing the reader directly to the tables which contain added details.

**Discussion:**

- The discussion has been edited to significantly reduce the word count and deliver a more concise appraisal of the results in the context of the current literature as suggested. As a result, the comments associated with particular lines of text have not been responded to individually as they have been edited as part of the overall process.
- Based on the comments associated with the “Practical Applications” section, no amendments have been made to this section.

We look forward to hearing from you in due course.

Best wishes,

Dr James W Bray

On behalf of all authors
