# Supplementary material for: Creatine kinase and neuromuscular fatigue responses following differing spells of simulated cricket fast bowling
Source: PLoS One. 2025 Jan 22;20(1):e0317692. doi: 10.1371/journal.pone.0317692 (PMC11753691; doi:10.1371/journal.pone.0317692)
Supplement: Attachment — Submitted filename: Response to Reviewers V2 Rebuttal (Nov).docx [file pone.0317692.s004.docx]

19^th^ November 2024

PONE-D-24-16732R1; Creatine Kinase and Neuromuscular Fatigue Responses Following Differing Spells of Simulated Cricket Fast Bowling

Dear Reviewer(s),

As per our earlier correspondence, we would like to thank you again for reviewing our revised research article for consideration for publication within the ‘PLOS ONE’ journal.

In this document (below), we address each of the points raised, providing specific details on where changes have been made. Where a change has not been made, we offer a clear justification as to why we believe it is not warranted.

**Point 1:**

- In my opinion the sample size (n=11) is simply too low to draw general conclusions. This fact is even more aggravated by the level of play of the participants, who do not present the elite population of the athletes in the sport. Therefore, the findings cannot be generally accepted for high level cricketers.

Our response

While it has been suggested that the current sample size (*n* = 11) and level of play, insufficient to draw general conclusions, we respectfully disagree. Firstly, in line with other team sports, cricket players have distinct roles within the team structure. Specifically, fast bowlers typically constitute three to five of the 11 players in a cricket team, irrespective of level of play (McNamara, Gabbett, Chapman, Naughton & Farhart, 2015a). Thus, recruiting 11 fast bowlers for this study effectively represents the fast-bowling contingents of approximately three different teams. Additionally, as outlined in our Methods section (lines 145 to 147), we have acknowledged the sample size limitation and provided context by citing similar studies with comparable sample sizes. Numerous previous investigations (see in-text citations, line 147) on simulated fast bowling have employed comparable sample sizes, further supporting the validity of our approach.

Secondly, regarding the level of play, we consider recruiting senior-level, club fast-bowlers a valid recruitment approach. Several studies have also used similar descriptive language when describing their fast bowlers. For instance, Feros, Young and O’Brien (2018) refer to their fast bowlers as ‘community-standard’. Similarly, McNamara, Gabbett, Naughton, Farhart, and Chapman (2013) describe their fast bowlers as ‘highly motivated’ competing for their individual clubs – terminology that is open to interpretation. Moreover, both McNamara, Gabbett, Chapman, Naughton and Farhart (2015b) and Minett, Duffield, Kellett and Portus (2012) describe their fast bowlers as ‘well-trained, highly skilled, senior club-level,’ language closely aligned with that used in our study.

Finally, we acknowledge in our manuscript that physical differences may exist between professional and club-level fast bowlers. We have carefully noted that further research is warranted to investigate the influence of playing standard on neuromuscular fatigue (NMF), avoiding sweeping statements that link our data to elite cohorts.

References

Feros, S. A., Young, W. B., & O’Brien, B. J. (2018). The reliability and sensitivity of performance measures in a novel pace-bowling test. International Journal of Sports Physiology and Performance, 13(2), 151-155.

McNamara, D. J., Gabbett, T. J., Chapman, P., Naughton, G., & Farhart, P. (2015a). The validity of microsensors to automatically detect bowling events and counts in cricket fast bowlers. *International Journal of Sports Physiology and Performance, 10*(1), 71-75.

McNamara, D. J., Gabbett, T. J., Chapman, P., Naughton, G., & Farhart, P. (2015b). Variability of PlayerLoad, bowling velocity, and performance execution in fast bowlers across repeated bowling spells. *International Journal of Sports Physiology and Performance*, *10*(8), 1009-1014.

McNamara, D. J., Gabbett, T. J., Naughton, G., Farhart, P., & Chapman, P. (2013). Training and competition workloads and fatigue responses of elite junior cricket players. *International Journal of Sports Physiology and Performance, 8*(5), 517-526.

Minett, G. M., Duffield, R., Kellett, A., & Portus, M. (2012). Mixed-method pre-cooling reduces physiological demand without improving performance of medium-fast bowling in the heat. *Journal of Sports Sciences, 30*(9), 907-915.

**Point 2:**

- CMJ jump is possibly not the best option for measuring fatigue for this sport as lower body movement contributes a much lower portion to the performance, compared to other team sports, such as football. Especially jump height is not the most informative parameter for neuromuscular fatigue, because jumping technique can be altered. For this please review more thoroughly the relevant literature.

Our response

While it has been suggested that a CMJ test may not be the most suitable option for quantifying fatigue in this sport, we respectfully disagree. The fast-bowling action involves repeated high-intensity episodes of upper- and lower-body accelerations and decelerations (Johnstone & Ford, 2010). As a result, fast bowlers experience significant ground reaction forces (5-9 times body weight), which necessities substantial eccentric strength in the quadriceps to withstand these repetitive forces (Johnstone & Ford, 2010). Moreover, Pyne, Duthie, Saunders, Petersen and Portus (2006), have also identified lower-body strength as a critical factor in determining delivery velocity. This is primarily based on the principle of proximal to distal sequencing, and the concept of efficient energy transfer up the mechanical chain. Numerous studies have demonstrated that ball release speed is impacted by run up speed, peak force development and loading rate (Ramachandran, Singh, Connor and Doma, 2024). These factors are all associated with power production from the lower limbs. Given this, we maintain that lower-body power, as assessed through a CMJ test, is an appropriate and valid measure for monitoring fatigue in fast bowlers. This position is further substantiated by several studies on fast bowling (Pote, Proctor, McEwan, Davy, & Christie, 2019; McNamara, Gabbett, Naughton, Farhart & Chapman, 2013; Johnstone & Ford, 2010), which have consistently employed CMJ tests as a reliable method to quantify fatigue in this population.

References

Johnstone, J. A., & Ford, P. A. (2010). Physiologic profile of professional cricketers. *The Journal of Strength and Conditioning Research*, *24*(11), 2900-2907.

Johnstone, J. A., Mitchell, A. C., Hughes, G., Watson, T., Ford, P. A., & Garrett, A. T. (2014). The athletic profile of fast bowling in cricket: A review. *The Journal of Strength and Conditioning Research, 28*(5), 1465-1473.

McNamara, D. J., Gabbett, T. J., Naughton, G., Farhart, P., & Chapman, P. (2013). Training and competition workloads and fatigue responses of elite junior cricket players*. International Journal of Sports Physiology and Performance, 8*(5), 517-526.

Pote, L., Proctor, S., McEwan, K., Davy, J. P., & Christie, C. J. (2019). The impact of a fast bowling spell on physiological, perceptual and performance responses in non-elite cricketers. *South African Journal of Sports Medicine, 31*(1), 1-5.

Pyne, D. B., Duthie, G. M., Saunders, P. U., Petersen, C. A., & Portus, M. R. (2006). Anthropometric and strength correlates of fast bowling speed in junior and senior cricketers. *The Journal of Strength and Conditioning Research, 20*(3), 620-626.

Ramachandran, A. K., Singh, U., Connor, J. D., Doma, K. (2024) Biomechanical and physical determinants of bowling speed in cricket: a novel approach to systematic review and meta-analysis of correlational data. *Sports Biomechanics*, *23*(3), 347-369.

**Point 3:**

- Also Player Load might not be most appropriate metric for measuring load on players. It largely correlates with distance traveled, which is obviously lower in cricket than in other team sports. Therefore, PlayerLoad might not provide the entire picture regarding the load.

Our response

While it has been suggested that PlayerLoad may not be the most appropriate metric for measuring load on players, due to its association with distance covered we disagree with this point. As outlined in our response to **Point 2** (see above), the fast-bowling action involves repeated high-intensity episodes of upper- and lower-body accelerations and decelerations (Johnstone & Ford, 2010). This action is characterised by inherent variability in bowling technique, despite the rules and regulations to standardise the bowling action (McNamara, Gabbett, Chapman, Naughton & Farhart, 2015). Given this, acceleration-based wearable technology, specifically microelectromechanical systems (MEMS) utilising tri-axial accelerometery, provides a comprehensive assessment of movement across anterior-posterior, medial-lateral, and vertical planes. Such devices are well-suited to capture key sport-specific activities (i.e., the fast bowling action) including accelerations, decelerations and jumps (Nicolella, Torres-Ronda, Saylor & Schelling, 2018). PlayerLoad, derived from tri-axial accelerometery, has become a widely adopted metric for monitoring workloads across various team sports (Cummins, Orr, O’Connor & West, 2013; Kempton, Sirotic & Coutts, 2014).

In the context of cricket, specifically fast bowling, PlayerLoad has been shown to be an appropriate measure, with research demonstrating its stability across bowling spells, reinforcing its utility for load monitoring in this context (McNamara, Gabbett, Chapman, Naughton & Farhart, 2015).

The fast-bowling action involves repeated high-intensity episodes of upper- and lower-body accelerations, with variations influenced primarily by the bowling restrictions of different match formats. For instance, limited-overs cricket imposes upper thresholds of 4-overs per bowler in Twenty20 matches, and 10-overs in One-Day matches. Match-play data (Petersen, Pyne, Dawson, Portus & Kellett, 2010) highlights that, among all positions, fast bowlers operate at the highest intensities across formats, covering 20-80% greater distances (format dependant). Notably, Petersen and colleagues (2010) also found that fast bowlers typically engage in repeated bouts of high-speed running almost exclusively during bowling spells.

To better understand the fatigue associated with fast bowling, field-based protocols have been developed to simulate the activity patterns associated with a spell of fast bowling. Simulations offer controlled and reproducible environments, allowing for the precise evaluations of match-play characteristics (Brito, Costa, Figueiredo & Brito, 2022). Based on these considerations, we used an iteration of the Cricket Australia-Australian Institute of Sport (CA-AIS) fast bowling skills test. This approach ensured that load was standardised to account primarily for the fast bowling activity itself while minimising the inherent variability associated in match-play.

References

Brito, P., Costa, J. A., Figueiredo, P., & Brito, J. (2022). Simulated Soccer Game Protocols: A Systematic Review on Validated Protocols That Represent the Demands of the Game. *The Journal of Strength and Conditioning Research, 38*(1), 192-205.

Cummins, C., Orr, R., O’Connor, H., & West, C. (2013). Global positioning systems (GPS) and microtechnology sensors in team sports: a systematic review. *Sports Medicine*, *43*, 1025-1042.

Duffield, R., Carney, M., & Karppinen, S. (2009). Physiological responses and bowling performance during repeated spells of medium-fast bowling. *Journal of Sports Sciences*, *27*(1), 27-35.

Johnstone, J. A., & Ford, P. A. (2010). Physiologic profile of professional cricketers. *The Journal of Strength and Conditioning Research*, *24*(11), 2900-2907.

Kempton, T., Sirotic, A. C., & Coutts, A. J. (2014). Between match variation in professional rugby league competition. *Journal of Science and Medicine in Sport*, *17*(4), 404-407.

McNamara, D. J., Gabbett, T. J., Chapman, P., Naughton, G., & Farhart, P. (2015). Variability of PlayerLoad, bowling velocity, and performance execution in fast bowlers across repeated bowling spells. *International Journal of Sports Physiology and Performance*, *10*(8), 1009-1014.

Nicolella, D. P., Torres-Ronda, L., Saylor, K. J., & Schelling, X. (2018). Validity and reliability of an accelerometer-based player tracking device. *PloS One*, *13*(2), e0191823.

Petersen, C. J., Pyne, D., Dawson, B., Portus, M., & Kellett, A. (2010). Movement patterns in cricket vary by both position and game format. *Journal of Sports Sciences*, *28*(1), 45-52

**Point 4:**

- Similar findings apply for the creatine kinase. Its response is highly individual specific and can be affected by many factors. However, CK elevation might not be a result of the lower body action, but the arm movement, which has a much lower amount of muscle mass. In the dataset there are also obvious outliers which should be exclude, because largely affects the statistical process, thanks to the low sample size. In most cases we do not see significant increases in CK level of participants.

Our response

While it has been suggested that creatine kinase (CK) levels are highly individual and may be generated primarily through arm movement rather than lower body activity, we respectfully disagree. Firstly, we would like to remind the reviewers of the complete fast bowling action. As previously described in **Point 2** (see above), the fast-bowling action involves repeated high-intensity episodes of upper- and lower-body accelerations and decelerations (Johnstone & Ford, 2010). Moreover, Law 26 (MCC, 1976) states that the ball is deemed to be thrown if any straightening of the bowling arm occurs during the delivery swing, prior to the ball leaving the hand (Bartlett, Stockill, Elliott & Burnett, 1996). Consequently, this regulation restricts the bowling arm to circumduction, thereby resulting in minimal eccentric loading of the arm muscles. Eccentric exercise, which forcibly lengthens muscles, is widely accepted as a primary contributor to muscle damage, muscle membrane disruption, reductions in maximal strength, and muscle soreness – all of which can lead to the leakage of muscle proteins, including CK (Clarkson, Nosaka & Braun, 1992; Schoenfeld, 2012).

In the context of team sport, CK is a well-established biomarker for quantifying muscle damage resulting from training and competition (McLellan, Lovell & Gass, 2011; Hunkin, Fahrner & Gastin, 2014). While we acknowledge that CK values at rest and following exercise can vary significantly between individuals (Kim & Lee, 2015), as reflected in our data, these differences are influenced by training status and mode of exercise. However, the suggestion by the reviewer to remove obvious outliers would contradict one of the key criteria outlined by PLOS ONE, which emphasises the importance of compliance with ethical standards (PLOS ONE, 2024). Instead, we have included 95% confidence intervals (95% CIs) to clearly demonstrate the precision of our estimates and to mitigate concerns about potential publication bias (Mesquida, Murphy, Lakens & Warne, 2022).

Collectively, these studies, combined with the biomechanics of the fast-bowling action, emphasise the significant contribution of the lower-body to overall muscle damage. Several studies (McLellan, Lovell & Gass, 2011; Schoenfeld, 2012; Hunkin, Fahrner & Gastin, 2014; Kim & Lee, 2015), specifically identify increases in CK following exercises with an eccentric component. Finally, throughout our manuscript we have carefully tempered our interpretation of CK data to ensure that it neither overemphasises its importance nor unduly influences the conclusions of practitioners and/or readers.

References

Bartlett, R. M., Stockill, N. P., Elliott, B. C., & Burnett, A. F. (1996). The biomechanics of fast bowling in men's cricket: A review. *Journal of Sports Sciences*, *14*(5), 403-424.

Clarkson, P. M., Nosaka, K., & Braun, B. (1992). Muscle function after exercise-induced muscle damage and rapid adaptation. *Medicine and Science in Sports and Exercise*, *24*(5), 512-520.

Elston D. M. (2021). Cherry picking, HARKing, and P-hacking. *Journal of the American Academy of Dermatology*, S0190-9622(21)01985-X

Hunkin, S. L., Fahrner, B., & Gastin, P. B. (2014). Creatine kinase and its relationship with match performance in elite Australian Rules football. *Journal of Science and Medicine in Sport*, *17*(3), 332-336.

Johnstone, J. A., & Ford, P. A. (2010). Physiologic profile of professional cricketers. *The Journal of Strength and Conditioning Research*, *24*(11), 2900-2907.

Kim, J., & Lee, J. (2015). The relationship of creatine kinase variability with body composition and muscle damage markers following eccentric muscle contractions. *Journal of Exercise Nutrition and Biochemistry*, *19*(2), 123-129.

Marylebone Cricket Club (1976). *The M.C.C. Cricket Coaching Book.* London: William Heinemann.

McLellan, C. P., Lovell, D. I., & Gass, G. C. (2011). Biochemical and endocrine responses to impact and collision during elite rugby league match play. *The Journal of Strength and Conditioning Research*, *25*(6), 1553-1562.

Mesquida, C., Murphy, J., Lakens, D., & Warne, J. (2022). Replication concerns in sports and exercise science: a narrative review of selected methodological issues in the field. *Royal Society open science*, *9*(12), 220946.

PLOS ONE (2024). *Journal Information. Why Researchers Choose PLOS ONE.* <https://journals.plos.org/plosone/s/journal-information#loc-open-access>

Schoenfeld, B. J. (2012). Does exercise-induced muscle damage play a role in skeletal muscle hypertrophy? *Journal of Strength and Conditioning Research 26*(5), 1441-1453.

**Point 5:**

- Even though, the paper is carefully prepared, it still contains some fundamental flaws which I would suggest to change. First of all, a satisfying sample size with real life/match situations would be necessary to detect actual changes. Load parameters, such as PlayerLoad and CMJ metrics should be carefully chosen after thoroughly browsing the literature. Limiting factors should be minimised, if not possible, than provide the in the appropriate section.

Our response

We appreciate the reviewers constructive feedback and acknowledgement that our paper has been carefully prepared. However, we respectfully disagree with the points pertaining to sample size, the use of PlayerLoad and CMJ, for reasons specifically articulated throughout this rebuttal (see relevant points above).

With regard to CK, we acknowledge that CK responses can vary widely between individuals due to a myriad of factors (Kim & Lee, 2015). Nonetheless, our inclusion of confidence intervals ensures that such variability is appropriately accounted for in the interpretation of results, providing robust and transparent representation of the data. This approach is in alignment with ethical guidelines for data reporting and helps minimise potential biases (Mesquida, Murphy, Lakens & Warne, 2022).

That said, we recognise that previous version(s), of this manuscript may not have fully addressed some of the potential limitations, particularly concerning CK variability (see **Point 4**). In light of this, we have revised the discussion section to explicitly include these limitations and provide greater context for the reader (P21, L418-426). We believe this addition further strengthens the integrity and clarity of our study.

Best wishes,

Dr James W Bray

On behalf of all authors
