# Supplementary material for: Creatine kinase and neuromuscular fatigue responses following differing spells of simulated cricket fast bowling
Source: PLoS One. 2025 Jan 22;20(1):e0317692. doi: 10.1371/journal.pone.0317692 (PMC11753691; doi:10.1371/journal.pone.0317692)
Supplement: Attachment — Submitted filename: Reviewer comments.pdf [file pone.0317692.s002.pdf]

Review: Creatine Kinase and Neuromuscular Fatigue Responses Following Differing Spells of Simulated Cricket Fast Bowling

Overall:

The authors should be commended on an admirable manuscript examining an area that is unique and under researched. A wide range of relevant literature has been used showing the authors are well read in the topic of interest and understand the game of cricket (as per the protocol in the methods). However, prior to publication there are some areas of concern that need to be addressed. While overall, the manuscript is really well written, as it stands it reads like a thesis rather than a journal article which needs to get key findings across in a concise manner. I have added more specific comments based on this below. If the manuscript can be re-worked taking into account this consideration, I feel it will be publishable in the target journal.

Abstract:

P2L32: Grammatical error: "aimed assess"

Player load needs to come in earlier in your abstract. It is mentioned in the results, but the reader is not sure what this means as it has not been added to the methods.

Introduction:

While really well written, it is quite long and descriptive in certain paragraphs i.e., definitions and explanations and stating the findings of each study one-by-one instead of integrating them together. By re-working, re-writing these sections it will make the introduction short, concise and to the point while still covering the key aspects of your investigation. It almost reads as a thesis as it stands rather than a journal article. This could also be done by adding references at the end of the sentences instead of within. You have all the information and are well read in the topic of interest, but it is too long winded as it stands.

P3L56-57: Would consider removing "accounting for three to five….." Not sure this is needed.

P3L64-69: Break into two sentences-it is difficult to follow as it is written.

Methods:

P7L159: What makes these bowlers highly skilled?

Please change “Subjects” to “Participants”

A lot of what you have discussed in your design section should actually be in a separate “Procedures section”. Please break into two.

P10L226-233: I feel this needs to come into the methods earlier on. I was quite confused as to what the CA-AIS was until I got to this paragraph.

I take it your data was normally distributed as you ran an ANOVA instead of a Kruskal Wallis test? The same as a t-test instead of a Wilcoxon signed-rank test? If this is the case then it needs to be stated in your data analysis section.

Overall, this section is well written, and it would be easy to reliably replicate the study based on the information provided.

Results:

This section contains all the relevant results from the investigation which are really interesting. However, I feel that a lot of what is displayed in the tables and figures is repeated in-text; please could you re-work so this is not the case. Please could you also check spacings in your tables- as it stands, they are very busy and quite difficult to make sense of due to uneven spacings.

Discussion

P17L356-358: not needed as the reader already knows what the purpose of the study is.

P17L374-376: this is repetitive of above.

As per my comment for the introduction, I think this section reads like a thesis rather than a research paper. There is quite a bit of repetition of the results and long explanations of different methodologies and studies that have been conducted previously. While it is important to acknowledge these methodological flaws and differences, the discussion needs to be short and concise with a critical analysis of the relevant literature. Please re-write/re-work.

Practical applications:

Excellent practical applications section.

References:

Wide range of relevant literature has been used.
